# Incessant ventricular tachycardia

**DOI:** 10.1007/s12471-016-0877-8

**Published:** 2016-08-25

**Authors:** R. Prisecaru, L. Riahi, Y. de Greef, D. Stockman, B. Schwagten

**Affiliations:** Cardiovascular Centrum Middelheim, Antwerpen, Belgium

A 65-year-old patient with paroxysmal atrial fibrillation and a normal structural heart underwent cryoballoon pulmonary vein isolation (PVI) under general anaesthesia. After PVI, adenosine 12 mg was given as a rapid intravenous bolus to test each pulmonary vein and no dormant sleeves were documented. The transseptal sheath was withdrawn into the right atrium and afterwards from the right groin. Thereafter, the patient developed pulseless monomorphic ventricular tachycardia (Fig. 1). Subsequently, intravenous injection of amiodarone and a β blocker were administrated and repeated cardiac defibrillations were attempted, but without sustained restoration of sinus rhythm. Inferior ST elevation was also documented.

**Fig. 1:**
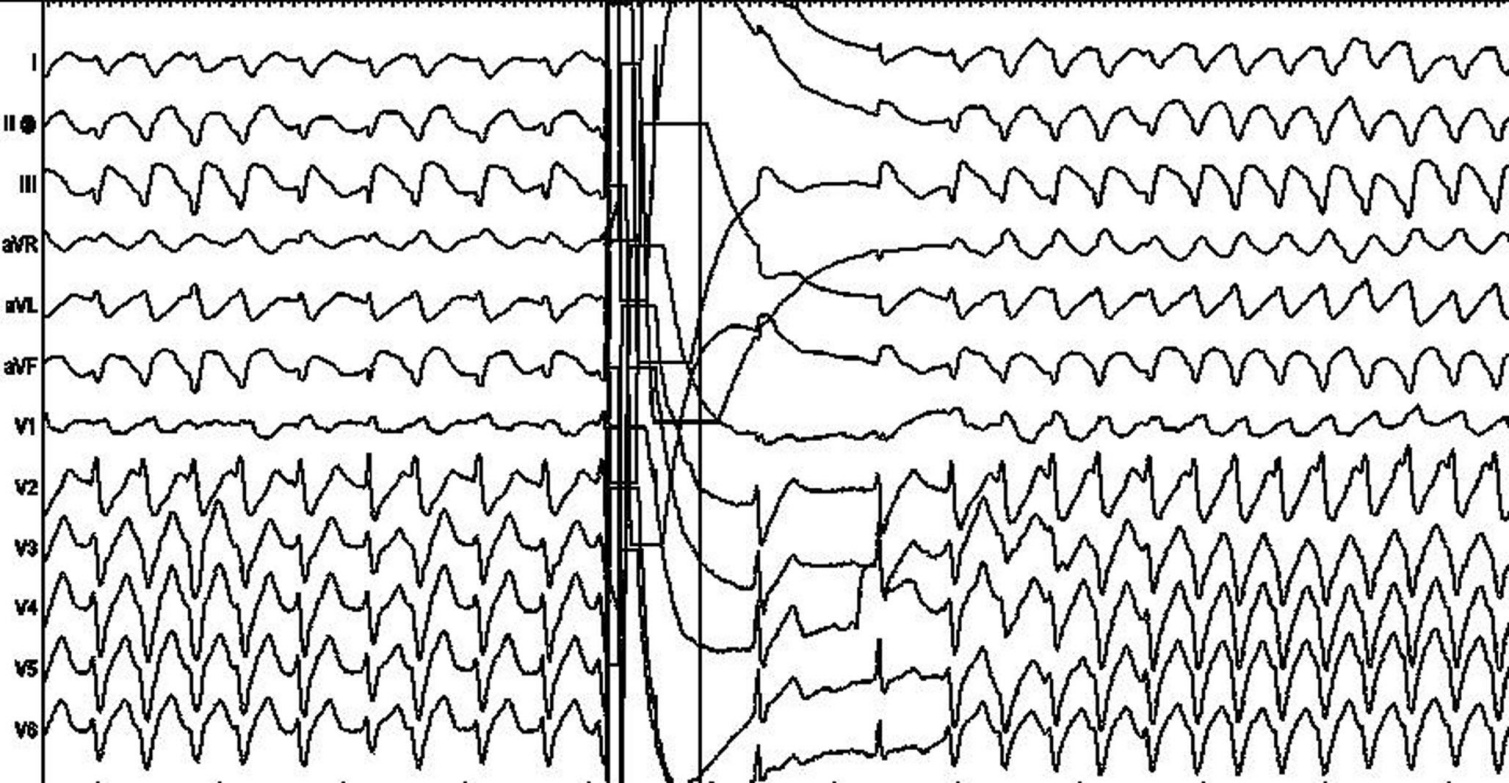
Twelve-lead ECG revealing monomorphic ventricular tachycardia, followed by successful electric cardioversion and thereafter return to ventricular tachycardia

Coronary angiography revealed diffuse coronary vasospasm (Fig. 2). After intracoronary nitrate infusion, resolution of the coronary vasospasm, complete cessation of the ventricular tachycardia and normalisation of the ST segment were noticed.

**Fig. 2:**
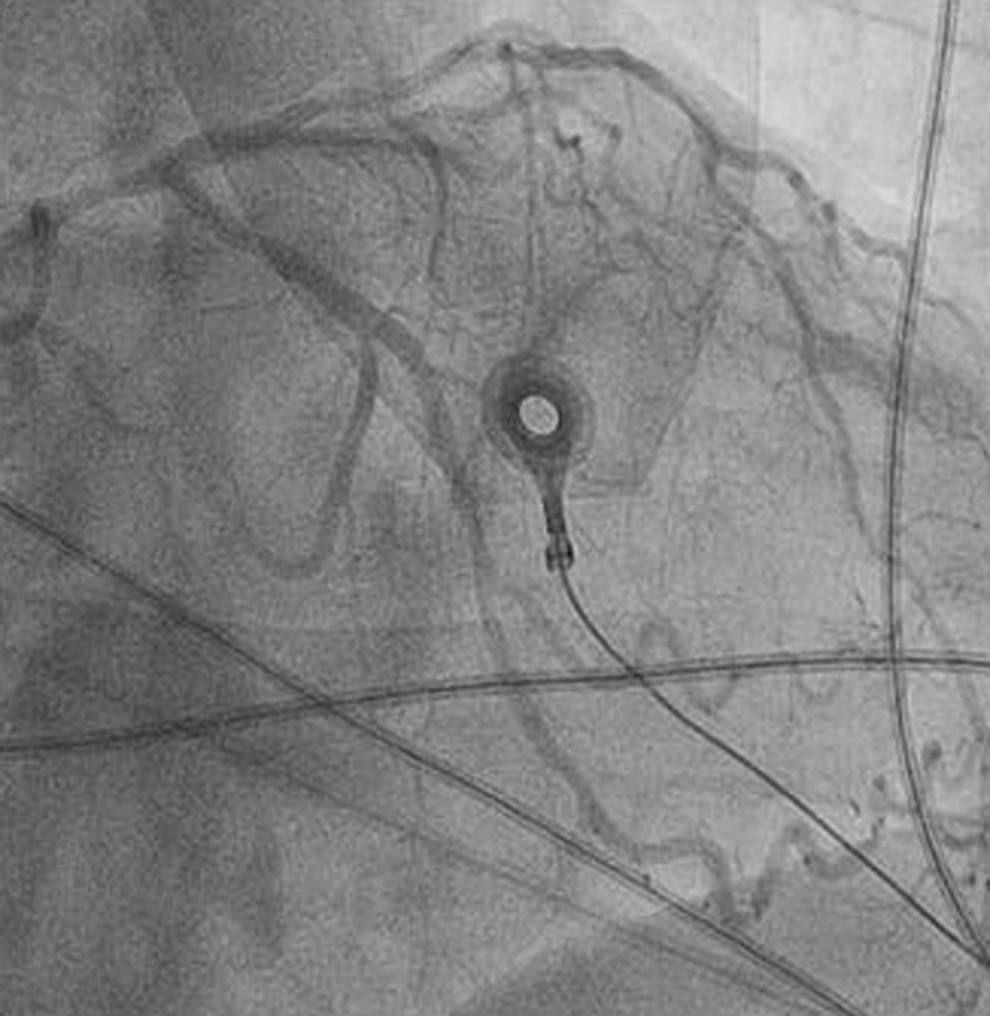
Left coronary angiography showing diffuse vasospasm

Question: What is the underling mechanism of the ventricular tachycardia from Fig. 1?

## Answer

You will find the answer elsewhere in this issue.

